# Monitoring specific antibody responses against the hydrophilic domain of the 23 kDa membrane protein of *Schistosoma japonicum *for early detection of infection in sentinel mice

**DOI:** 10.1186/1756-3305-4-172

**Published:** 2011-09-10

**Authors:** Jie Wang, Chuan-Xin Yu, Xu-Ren Yin, Wei Zhang, Chun-Yan Qian, Li-Jun Song, Xue-Dan Ke, Yong-Liang Xu, Wei He, Guo-Qun Cao

**Affiliations:** 1 Jiangsu Institute of Parasitic Diseases, Wuxi 214064, People's Republic of China

## Abstract

**Background:**

Schistosomiasis remains an important public health problem throughout tropical and subtropical countries. Humans are infected through contact with water contaminated with schistosome cercariae. Therefore, issuing early warnings on the risk of infection is an important preventive measure against schistosomiasis. Sentinel mice are used to monitor water body infestations, and identifying appropriate antibody responses to schistosome antigens for early detection of infection would help to improve the efficiency of this system. In this study we explored the potential of detecting antibodies to the hydrophilic domain (HD) of the 23-kDa membrane protein (Sj23HD) and soluble egg antigen (SEA) of *Schistosome japonicum *for early detection of schistosome infection in sentinel mice.

**Results:**

Development of IgM and IgG antibody levels against Sj23HD and SEA in *S. japonicum *infected mice was evaluated over the course of 42 days post-infection by enzyme-linked immunosorbent assay (ELISA) and immunoblotting. The Sj23HD and SEA specific IgM and IgG levels in mice all increased gradually over the course of infection, but IgM and IgG antibodies against Sj23HD presented earlier than those against SEA. Furthermore, the rates of positive antibody responses against Sj23HD were higher than those against SEA in the early stage of schistosome infection, suggesting that the likelihood of detecting early infection using anti-Sj23HD responses would be higher than that with anti-SEA responses. The use of immunoblotting could further improve the early detection of schistosome infection due to its greater sensitivity and specificity compared to ELISA. Additionally, the levels of Sj23HD and SEA specific antibodies positively correlated with the load of cercariae challenge and the duration of schistosome infection.

**Conclusions:**

This study demonstrated that antibody responses to the Sj23HD antigen could be monitored for early detection of schistosome infection in mice, especially by immunoblotting which demonstrated greater sensitivity and specificity than ELISA for detection Sj23HD antibodies.

## Background

Schistosomiasis is an important tropical parasitic disease, with more than 200 million people currently infected among the 779 million people at risk of infection worldwide [1]. In the People's Republic of China (P.R. China), schistosome infection mainly occurs in the marshland and lake regions of Hunan, Hubei, Jiangxi, Anhui and Jiangsu provinces and in the hilly and mountainous regions of Sichuan and Yunnan provinces where the interruption of schistosomiasis transmission has been proven particularly difficult to achieve [2]. At present, approximately 65 million individuals are still at risk of infection in eastern Asia, including P.R. China [3,4], despite significant efforts to control the disease over the past 60 years [5].

Schistosome infections generally peak during the flood season (from May to October) along the Yangtze River, especially in the middle and lower reach of the Yangtze River [6-8]. Humans become infected in P.R. China mainly through contact with water infested with *Schistosome japonicum *cercariae [9]. Reducing the incidence of infection remains an ongoing aim for schistosomiasis control. Identifying infested water areas, issuing timely infection risk warnings as well as instituting intervention measures are helpful for preventing infection and controlling the prevalence of schistosomiasis. Traditionally, sentinel mice are used to monitor schistosome infested water bodies [10]. However, the maturation of schistosomes from schistosomula to adult worms takes approximately 22 days [11], and relying on counting worm burden to determine infection in sentinel mice makes it difficult to provide early warnings to people on the risk of schistosome infection for any given infested water body.

Fortunately, schistosome antibodies (IgM or IgG) to schistosome antigens present in the serum of the host within 1-2 weeks post-infection [12] and, therefore, would be more efficient as markers of infection than adult worms in sentinel mice. Although the presence of the circular antigen of the schistosome would better reflect the infection status of a host [13] than that of the antigen-specific antibody response, the efficiency of existing detection methods for the circular antigen are low and cannot be used for diagnosis of schistosomiasis [14,15]. A series of tests for detecting schistosome specific antibodies have been developed, such as the cercarien-huellen reaction (CHR), circumoval precipitin test (COPT), enzyme-linked immunosorbent assay (ELISA), indirect hemagglutination assay (IHA) and dipstic dye immunoassay (DDIA) [16]. These methods are commonly used for diagnosis and surveillance of schistosome infection, but none of them can be adequately used for early detection. As the protein expression profiles in different developmental stages of schistosome are different, the antibodies against schistosomula antigens should present first in the sera of sentinel mice after infection and may be used as potential markers for early diagnosis of schistosome infection. It has been shown that the 23 kDa membrane protein of *S. japonicum *(Sj23) exists in all stages of the parasite but the egg and is notably detected in the lung stage [17]. Sj23 plays an important role in maintaining growth and development of *S. japonicum *and is of interest as a potential vaccine candidate [18]. Therefore, detecting antibody responses to the Sj23 protein may be promising for early diagnosis of schistosome infection.

To develop a method for early detection of *S. japonicum *infection in sentinel mice, the dynamics of specific IgM and IgG antibodies responses to the hydrophilic domain (HD) of the Sj23 membrane protein (Sj23HD) and soluble egg antigen (SEA) in mice over the course of 42 days post-infection were systematically investigated in this study. These antibody levels were correlated with the load of cercariae used for infection and with the infection period. The efficiencies of ELISA and immunoblotting methods for detecting antibodies against Sj23HD and SEA were also compared.

## Methods

### Snails and cercariae

Snails (*Oncomelania hupensis*) infected with *S. japonicum *(typical schistosome species found in China) were provided by the Department of Snail Biology, Jiangsu Institute of Parasitic Diseases, Wuxi, P. R. China. *S. japonicum *cercariae were induced to hatch from infected snails by immersion in de-chlorinated water with illumination at 25°C for 2.5 h.

### Animals

ICR mice (female, 20 g, 6 weeks old) were purchased from the Experimental Animal Center of Yangzhou University, Yangzhou, P.R. China. Japanese white rabbits were provided by the Experimental Animal Facility of Nanjing General Hospital of Nanjing Military Command, Nanjing, P.R. China, and raised at the Department of Experimental Animal, Jiangsu Institute of Parasitic Diseases, Wuxi, P.R. China. The experiments were carried out with approval from the Animal Research Advisory Committee of the Jiangsu Institute of Parasitic Diseases.

### Reagents and instruments

Glutathione Sepharose 4B was purchased from GE Healthcare (Piscataway, NJ, USA). Ninety-six-well flat-bottomed ELISA plates were purchased from Greiner Bio-One Company (Frickenhausen, Germany). Bovine serum albumin (BSA) was obtained from BioDev-Tech. Co., Ltd. (Beijing, P.R. China). Horseradish peroxidase (HRP)-labeled goat anti-mouse IgM antibody and IgG antibody were purchased from Bethyl Laboratories (Montgomery, TX, USA). 3,3',5,5'-tetramethylbenzidine (TMB) substrate was purchased from Genescript Biotech Company (Piscataway, NJ, USA). Nitrocellulose membranes (0.22 μm) were purchased from Whatman (Piscataway, NJ, USA). Diaminobenzidine (DAB) was purchased from Bio Basic Inc. (Ontario, Canada). Skim milk was purchased from Becton, Dickinson and Company (Franklin Lakes, NJ, USA). The ELISA plate reader Anthos Zenyth 340 was manufactured by Biochrom Co., Ltd. (Cambridge, UK) and the Trans-Blot SD Semi-Dry Electrophoretic Transfer Cell by Bio-Rad (Hercules, CA, USA).

### Animal infection and serum collection

#### Experiment I

ICR mice were divided into a control group (n = 5) which received no treatment and an experimental group (n = 10) in which each animal was infected with 50 cercariae of *S. japonicum *by abdominal skin exposure [19]. Mouse sera were collected from the tail veins on days 7, 10, 14, 18, 21, 28, 35 and 42 post-infection and stored at -20°C for subsequent analysis. All mice were sacrificed on day 42 post-infection. The adult worms in the mesenteric veins and egg granuloma in the livers were surveyed to confirm that schistosome infections of the mice were successful.

#### Experiment II

ICR mice were divided into nine groups (A to I; n = 5 per group). Group A served as the control without any treatment. Mice in groups B to I (experimental groups) were infected with 5, 10, 15, 20, 25, 30, 35 and 40 cercariae of *S. japonicum*, respectively, by abdominal skin exposure. Mouse sera were collected on days 21 and 28 post-infection and stored at -20°C for subsequent analysis. All mice were sacrificed on day 42 post-infection. Schistosome infections of mice were confirmed as described for Experiment I.

### Preparation of antigens

The Sj23HD/pGEX-5X-1 plasmid expressing recombinant fusion protein (GST-HD) of the HD of the 23 kDa membrane protein and glutathione S-transferase (GST) of *S. japonicum *was constructed by our laboratory and transformed into *Escherichia coli *BL21 (DE3). The GST-HD fusion proteins expressed from the transformed *E. coli *were purified by using Glutathione Sepharose 4B based affinity chromatography following a previously described protocol [20]. *S. japonicum *SEA was prepared as a described elsewhere [21]. Briefly, Japanese white rabbits were infected with 1,500 cercariae by abdominal skin exposure and dissected on day 45 post-infection. The rabbit livers were homogenized in cold saline (1.2% sodium chloride), and the *S. japonicum *eggs were collected by filtrating the liver tissue homogenates through a 240 nylon mesh net. The liver tissues surrounding the egg surfaces were digested with typsin. The eggs were then washed with cold PBS and manually homogenized using a glass homogenizer. The egg homogenates were centrifuged at 25,000 × *g *at 4°C for 20 min. The supernatants containing SEA were collected, and the protein concentrations were determined using a BCA kit (Pierce Biotechnology, Inc., Rockford, IL, USA) and stored at -80°C.

### ELISA detection of *S. japonicum *antigen specific IgM and IgG antibodies in sera of infected mice

One hundred microliters of GST-HD or SEA proteins suspended in coating buffer (0.05 M sodium carbonate solution, pH 9.6) at a concentration of 10 μg/ml were added to each well of a microtiter plate and incubated at 4°C overnight. The wells were blocked for 1 h with 350 μl of PBS containing 0.05% Tween 20 (PBST) with 5% BSA at 37°C. After the plates were washed three times with PBST, 100 μl of serum diluted 1:100 in PBS containing 1% BSA were added to each well and incubated at 37°C for 1 h. After the plate was washed three times as above, the HRP-labeled goat anti-mouse IgM secondary antibody diluted 1:3,000 or IgG secondary antibody diluted 1:10,000 was added into each well, and the plate was incubated at 37°C for 1 h. The plates were then washed, and the reaction was developed by adding 100 μl of TMB substrate for 5 min in the dark at room temperature. The color development was stopped by adding 100 μl/well of 2 M sulfuric acid solution (H_2_SO_4_), and the optical density (OD) in each well was measured at 450 nm using an ELISA plate reader. The OD_450 _values of sample wells above 2.1 times that of negative control wells were judged as positive.

### Detection of *S. japonicum *specific IgM and IgG antibodies in sera of infected mice by immunoblotting

Approximately 100 μg of recombinant GST-HD fusion proteins or SEA were loaded on a 12% SDS-polyacrylamide gel with a strip format comb with 1 reference well (1.0 mm thick, Bio-Rad) and transferred onto a nitrocellulose membrane under constant voltage (20 V) for 30 min using a Trans-Blot Semi-Dry Electrophoretic Transfer Cell. The nitrocellulose membrane was blocked with 5% skimmed milk powder in PBST at room temperature for 1 h or at 4°C overnight. The blocked membrane then was cut longitudinally into strips of 3 mm width each. The strips were incubated for 1 h at room temperature with mouse sera (diluted 1:500) collected at different times post-infection. After three washes with TBST (10 mM Tris, 150 mM NaCl, PH 7.6, 0.05% Tween-20) (10 min each time), the strips were incubated with HRP-conjugated goat anti-mouse IgG (diluted 1:10,000) or HRP-conjugated goat anti-mouse IgM (diluted 1:3,000) for 1 h at room temperature. After washing the strips 3 times in TBST, the color reaction was developed by incubating the strips with DAB substrate for 2 min at room temperature and then terminated by washing the strips with distilled water.

### Statistical analysis of data

Values for antibody responses levels were compared and analyzed with SPSS statistical package for social sciences, version 13.0 software (Chicago, IL, USA). All data were expressed as means ± standard deviations.

## Results

### Dynamics of Sj23HD and SEA specific antibody responses detected by ELISA

**Figure 1:**
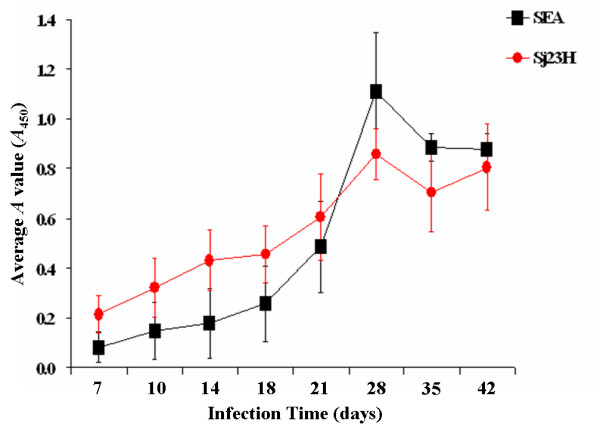
**Development of Sj23HD and SEA specific IgM antibodies in sera of *S. japonicum *infected mice over time detected by ELISA**. The IgM antibody responses to the Sj23HD antigen (circles) and SEA (squares) over the course of 42 days post-infection were detected by ELISA. The average antibody levels of Sj23HD or SEA specific IgM antibodies in sera on days 0, 7, 10, 14, 18, 21, 28, 35 and 42 post-infection respectively were indicated by means ± standard deviations of OD450 value of ten mice serum samples.

**Figure 2:**
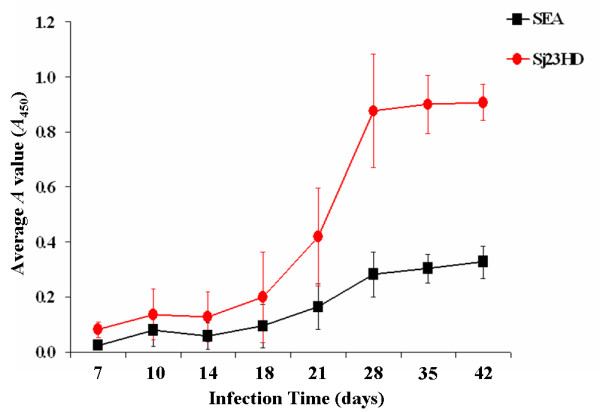
**Development of Sj23HD and SEA specific IgG antibodies in sera of *S. japonicum *infected mice over time detected by ELISA**. The IgG antibody responses to the Sj23HD antigen (circles) and SEA (squares) over the course of 42 days post-infection were detected by ELISA. The average antibody levels of Sj23HD or SEA specific IgG antibodies in sera on days 0, 7, 10, 14, 18, 21, 28, 35 and 42 post-infection respectively were indicated by means ± standard deviations of OD450 value of ten mice serum samples.

The Sj23HD and SEA specific IgM and IgG antibodies in the sera of mice were detected by ELISA and found to increase gradually over the course of infection. Sj23HD specific IgM appeared on day 10 post-infection, crossed the positive threshold on day 18 and then peaked on day 28 post-infection (Figure 1). Anti-SEA IgM first appeared on day 10 post-infection and then reached the positive threshold by day 21 and the peak value on day 28 post-infection. Thereafter, the levels of IgM antibodies against SEA gradually declined (Figure 1). Sj23HD specific IgG first appeared on day 14 post-infection, gradually increased on day 18, reached the positive threshold by day 21 and then elevated rapidly thereafter (Figure 2). Anti-SEA IgG first appeared on day 14 and reached the positive threshold on day 28 post-infection. Overall, the levels of IgG against SEA were lower than those against Sj23HD (Figure 2). Although the immune responses of individual mice to the schistosome antigens were obviously diverse, the Sj23HD specific IgM and IgG antibodies appeared earlier in mice than those specific for SEA (Table 1). These results demonstrated that anti-Sj23HD responses could potentially be used for early detection of schistosome infection.

**Table 1:** Comparison of the serologically positive rates of Sj23HD and SEA specific IgM and IgG antibodies in *S.japonicum *infected mice by ELISA

Time (days)	No.of mice	Specific IgM		Specific IgG	
	
		Sj23HD (%)	SEA(%)	Sj23HD(%)	SEA(%)
7	10	0	0	0	0
10	10	10	10	0	0
14	10	30	20	10	10
18	10	60	50	40	20
21	10	70	60	60	50
28	10	100	90	90	70

### Dynamics of SEA and Sj23HD specific IgG antibodies detected by immunoblotting

**Figure 3:**
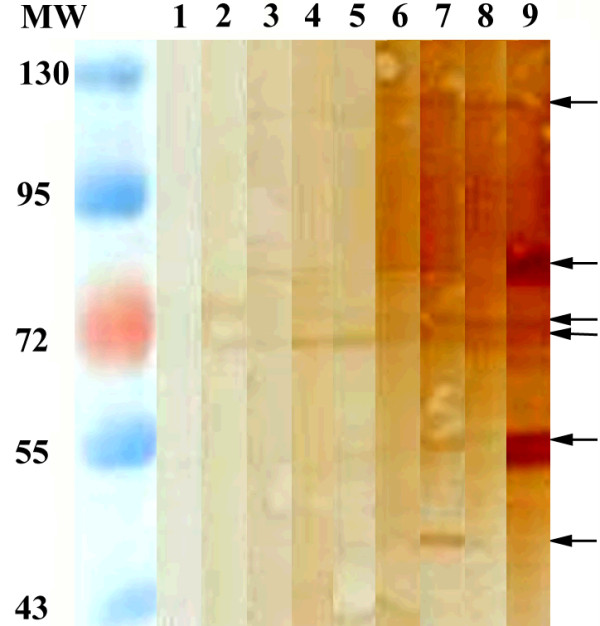
**Immunoblotting profile of SEA specific serum IgG of *S. japonicum *infected mice at different times post-infection**. Lane M, protein molecular weight markers; lane 1, SEA recognition by IgG in mixed sera of five mice before infection; lanes 2-9, SEA recognition by IgG in mixed sera of 10 mice at days 7, 10, 14, 18, 21, 28, 35 and 42 post-infection, respectively.

**Figure 4:**
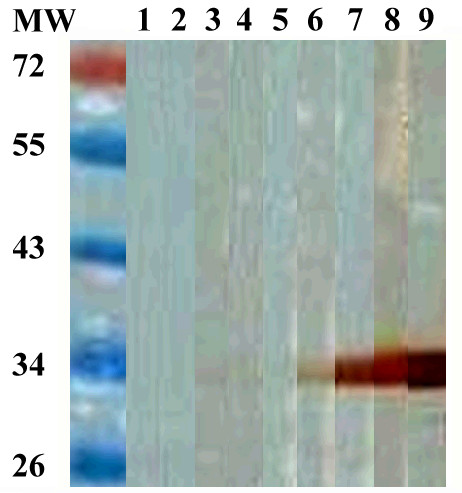
**Immunoblotting profile of Sj23HD specific serum IgG of *S. japonicum* infected mice at different times post-infection**. Lane M, protein molecular weight markers; lane 1, Sj23HD recognized by mixed sera of five mice before infection; lanes 2-9, Sj23HD recognition by IgG in mixed sera of 10 mice at days 7, 10, 14, 18, 21, 28, 35 and 42 post-infection, respectively.

The SEA and Sj23HD specific IgG in pooled sera of infected mice from day 0 to 42 were detected by immunoblotting. While no protein bands were recognized on day 0, the two SEA proteins of 73 and 78 kDa were recognized on day 7 post-infection. Recognition of the protein bands of 73, 78, 84, and 121 kDa occurred on day 14 post-infection. Five protein bands of 55, 73, 78, 84 and 121 kDa were recognized on day 18 post-infection. A 47 kDa protein band was also recognized on days 21 and 28 post-infection, but then this reactivity disappeared by day 35. The densities of the 55, 73, 78, 84 and 121 kDa protein bands continually enhanced over the infection time and peaked on day 42 (Figure 3). Similarly, the Sj23HD protein (expected size of 33.5 kDa) was specifically recognized early by the mouse serum IgG on day 7 post-infection, and this immune reactivity increased gradually over time, peaking on day 42 (Figure 4).

### Dynamics of SEA and Sj23HD specific IgM antibodies detected by immunoblotting

**Figure 5:**
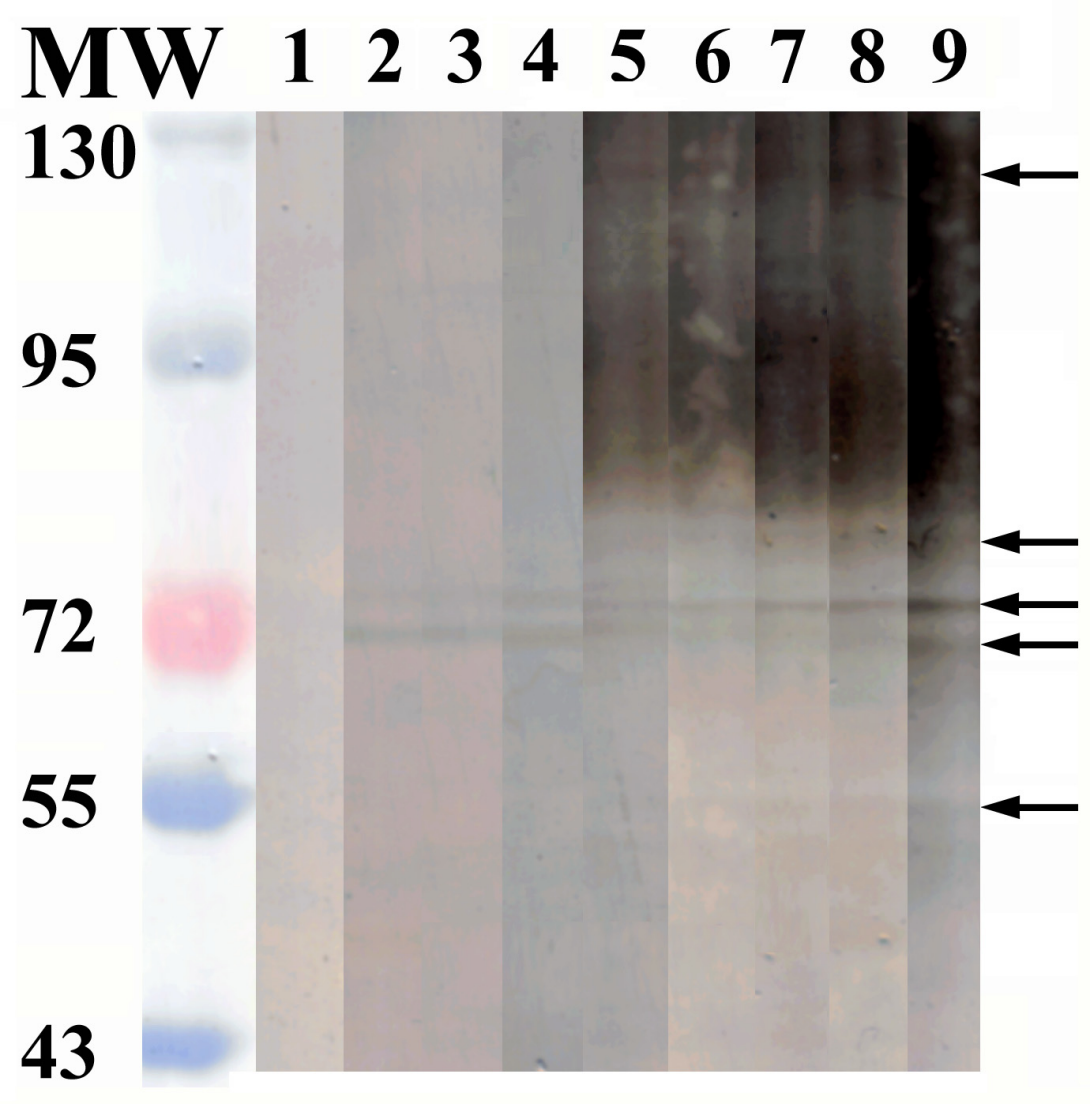
**Immunoblotting profile of SEA specific serum IgM of *S. japonicum *infected mice at different times post-infection**. Lane M, protein molecular weight markers; lane 1, SEA recognition by IgM in mixed sera of five mice before infection; lane 2-9, SEA recognition by IgM in mixed sera of ten mice at days 7, 10, 14, 18, 21, 28, 35 and 42 post-infection, respectively.

**Figure 6:**
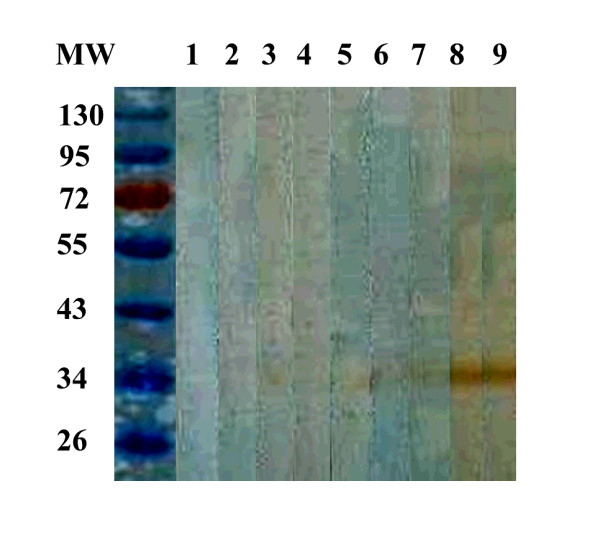
**Immunoblotting profile of Sj23HD specific serum IgM of *S. japonicum *infected mice at different times post-infection**. Lane M, protein molecular weight markers; lane 1, Sj23HD recognition by IgM of mixed sera of five mice before infection; lanes 2-9, Sj23HD recognition by IgM in mixed sera of 10 mice at days 7, 10, 14, 18, 21, 28, 35, 42 post-infection, respectively.

The SEA or Sj23HD specific IgM in pooled sera of infected mice from day 0 to 42 were detected by immunoblotting. The mouse sera did not recognize any protein bands on day 0, while the two SEA proteins of 73 and 78 kDa were recognized on day 7 post-infection. Five protein bands of 55, 73, 78, 84 and 121 kDa were recognized on day 18 post-infection. The intensities of the five protein bands continually enhanced over time and peaked on day 42 post-infection (Figure 5). The Sj23HD protein was also specifically recognized early by the mouse serum IgM on day 7 post-infection. This immunoreactivity also gradually enhanced as the infection time progressed and reached a peak on day 42 post-infection (Figure 6).

### Serologically positive rates of Sj23HD and SEA specific antibodies in *S. japonicum *infected mice detected by immunoblotting

The specific IgG and IgM antibody responses to Sj23HD of individual mice were detected by immunoblotting. The serum positive rates of Sj23HD specific IgG on days 0, 7, 10, 14, 18, 21 and 28 post-infection were 0, 10, 10, 30, 50, 80 and 100%, respectively; and the serum positive rates of Sj23HD specific IgM at the same times post-infection were 0, 10, 10, 30, 60, 90 and 100%, respectively (Table 2). The SEA specific IgG and IgM antibody responses of individual mice were also detected by immunoblotting. The serum positive rates of SEA specific IgG on days 0, 7, 10, 14, 18, 21 and 28 post-infection were 0, 20, 20, 30, 50, 70 and 90% respectively; and the serum positive rates of SEA specific IgM at the same times post-infection were 0, 10, 20, 30, 50, 70 and 100%, respectively (Table 2). The positive rates of Sj23HD specific antibodies at different times post-infection were higher than those of the SEA specific antibodies. Compared to the positive rates obtained by ELISA, the efficiency of detection by immunoblotting was significantly higher. (The immunoblotting profiles of serum reactivities against the SEA or Sj23HD protein bands of individual mice at different times post-infection are shown in Additional file 1 Figure S1; Additional file 2 Figure S2; Additional File 3 Figure S3 and Additional File 4 Figure S4.).

**Table 2:** Comparison of the serologically positive rates of anti-Sj23HD and anti-SEA IgM and IgG antibodies in the sera of mice infected with S. japonicum by immunoblotting

Time(days)	No.of mice	Anti-Sj23HD antibodies		Anti-SEA antibodies	
		IgM (%)	IgG(%)	IgM(%)	IgG(%)
0	5	0	0	0	0
7	10	10	10	10	20
10	10	10	10	20	20
14	10	30	30	30	30
18	10	60	50	50	50
21	10	90	80	70	70
28	10	100	100	100	90

### Correlation between the cercariae load used for infection and serologically positive rate of Sj23HD specific antibodies

To determine the relationship between antibody responses and the cercaria load used for infection, Sj23HD specific antibodies in mice infected with different numbers of cercariae were detected on days 21 and 28 by immunoblotting. As shown in Table 3 the positive rates of Sj23HD specific IgM and IgG on day 21 post-infection were 40% and 20% in mice infected with 5 cercariae (group B); 80% and 60% in mice infected with 10 cercariae (group C); both 80% in mice infected with 15 cercariae (group D); and 100% and 80% in mice infected with 20 cercariae (group E), respectively. At the same time, the positive rates of Sj23HD specific IgG and IgM were 100% in all mice infected with more than 20 cercariae (groups F-I). On day 28 post-infection, the positive rates of Sj23HD specific IgG and IgM were both 60% in mice infected with 5 cercariae (group B); and 100% and 80%, respectively, in mice infected with 10 cercariae (group C). Meanwhile, all groups infected with more than 15 cercariae (groups D-I) were 100% positive on day 28 for both Sj23HD specific IgG and IgM. These results indicated that the frequency of animals developing antibodies against Sj23HD were positively associated with the load of cercariae used for the challenge.

**Table 3:** Comparison of the serologically positive rates of anti-Sj23HD IgM and IgG in the sera of mice infected with different numbers of *S.japonicum *cercariae

Group	No. of cercariae	Anti-Sj23HD IgM		Anti-Sj23HD IgG	
	
		Day 21 (%)	Day 28 (%)	Day 21 (%)	Day 28 (%)
A	0	0	0	0	0
B	5	40	60	20	60
C	10	80	80	60	100
D	15	80	100	80	100
E	20	100	100	80	100
F	25	100	100	100	100
G	30	100	100	100	100
H	35	100	100	100	100
I	40	100	100	100	100

## Discussion

The current emphasis of schistosomiasis management in P.R. China is to survey areas where the transmission has been stopped and to control schistosomiasis epidemics in the areas where the prevalence of this parasitic infection has not been controlled effectively. Finding the susceptible, high risk environments for schistosome infection, issuing warnings of infection risk rapidly and taking more targeted measures for prevention and intervention to eliminate the incidence of infection are key goals for effective schistosomiasis control [22]. Therefore, developing an early diagnostic technique to meet the requirements of the current schistosomiasis prevention and control program is paramount [23,24].

The rapid development of modern genetic engineering techniques [25] makes it possible to isolate and produce specific antigens for detection of schistosome infections, and recombinant antigens can be used to improve the sensitivity and specificity of diagnostic methods. The recombinant Sj23 membrane protein has been shown to induce strong humoral immune responses and thus can be used as a diagnostic antigen to detect schistosome specific antibody responses [26,27]. Lu *et al*. used the recombinant large HD of Sj23 to diagnose *S. japonicum *infection in buffalo [28]. Yu *et al*. had used the recombinant fusion protein of GST and large HD of Sj23 (GST-HD) to diagnose *S. japonicum *infection in humans [21]. The Sj23 membrane protein is present in all stages of the schistosome life cycle [17]; therefore, Sj23 specific antibodies should theoretically appear in the early stage of schistosome infection and would be valuable for early clinical diagnosis, as well as for determining the infection status of sentinel mice.

In this study, the utilities of Sj23 and SEA, a widely used diagnostic antigen, were compared for early detection of schistosome infection in sentinel mice. The recombinant fusion protein GST-Sj23HD and SEA were used to detect specific serum IgM and IgG antibodies in *S. japonicum *infected mice at different times post-infection by ELISA and immunoblotting. The results showed that both anti-SEA and anti-Sj23HD IgM and IgG could be found at days 7 to 10 post-infection, and the antibody titers increased gradually over the course of infection. However, the titers of Sj23HD antibodies increased more quickly than those of the SEA specific antibodies. The positive rates of Sj23HD antibodies at different times post-infection were higher than those of the antibodies against SEA. At day 21 post-infection, the positive rates of Sj23HD specific IgG and IgM reached 80 and 90%, respectively, but the positive rates of both SEA specific IgG and IgM only reached 70%. These results suggested that the antibody responses against Sj23HD would be more valuable for early diagnosis of schistosome infection than those against SEA, which may be attributed to the fact that Sj23 is a dominant schistosomula antigen. We speculate that there are epitopes within SEA which can induce specific antibodies in the early stage of infection in mice; however, since these are non-dominant antigens in schistosomula, they are unable to induce high titer antibodies in the early stage of schistosome infection and result in only weak immuno-reactivity and a low positive rate of detection.

The specificity of an antibody detection method depends on the nature of the antigen used to capture the antibody. If the non-specific epitopes in the antigen are dominant, it would result in non-specific reactivity. In this study, the recombinant antigen Sj23HD was prepared from *E. coli*, and SEA was prepared from infected rabbit liver tissues, which could be contaminated with non-specific proteins. Thus, these pooled antigens used to detect antibodies by ELISA may result in cross-reactive or false positive responses. However, the immunoblotting assay can separate various components of the pooled antigens, and the specificity of a reaction can be evaluated based upon whether the serum antibodies recognize the protein band(s) of expected size. When using the purified GST-HD fusion protein, only the expected 33.5 kDa protein recognized by sera of mice post-infection could be considered as a positive response. For SEA, five main bands of 55, 73, 78, 84 and 121 kDa recognized by sera of mice post-infection could be judged as positive, especially the 73 and 78 kDa protein bands, which are present in nearly every stage of schistosome infection. Thus, the ability of the immunoblotting method to separate the antigens by size can help to avoid or reduce false positives due to non-specific protein reactions. For these reasons, the specificity of immunoblotting was determined to be higher than that of ELISA for detection of schistosome specific antibodies.

The results of this study also showed that the sensitivity of immunoblotting to detection of specific IgM and IgG was higher than that of ELISA, especially for detecting serum antibodies of mice in the early stage of infection. The lower detection efficiency of ELISA may be ascribed to the low titers of specific antibodies against schistosome antigens in the early stage of infection as well as the low antigen amounts coated on the wells of the microtiter plates. However, the amount of antigen transferred to the nitrocellulose membrane could be adjusted in accordance with the requirement of the immunoblotting conditions. For example, appropriately increasing the amount of antigen could be helpful in detecting low-titer schistosome specific serum antibodies. Therefore, the more efficient immunoblotting method would be more suitable than ELISA for early detection of schistosome infections.

Antibody levels are generally positively correlated with the amount and duration of antigen stimulation [29]. Indeed, the anti-Sj23 antibody levels in serum of schistosomiasis patients have been positively associated with the schistosome adult burden and infection duration [30-33]. Similarly, the results of this study also showed that the positive rates of anti-Sj23HD antibodies in mouse sera positively correlated with the load of *S. japonicum *cercariae used for the challenge and the duration of infection.

## Conclusions

This study demonstrated that the antibody responses to the 23 kDa membrane protein of *S. japonicum *are useful for early detection of schistosome infection in mice. Compared to the ELISA based method, immunoblotting using the recombinant Sj23HD antigen has improved sensitivity and specificity for detecting specific IgM or IgG antibodies.

## Competing interests

The authors declare that they have no competing interests.

## Authors' contributions

CXY designed and supervised the study and critically revised the manuscript. JW did the laboratory work, analysed the data, and drafted the manuscript. XRY expressed and purified recombinant GST-HD fusion protein. WZ participated in the immunoblotting laboratory work. LJS, CYQ and XDK contributed to data analysis. YLX prepared the serum of infected mice. WH and GQC prepared the SEA. All authors read and approved the final manuscript.

## Supplementary Material

Additional file 1 **Fig. S1 Immunoblotting profile of SEA recognition by serum IgG of 10 individual mice infected with *S. japonicum* at different times post-infection**. For all blots, lane M, protein molecular weight markers; lane 1, no protein band was recognized by pooled sera of five mice before infection; lanes 2-11, sera of 10 individual mice on the indicated days post-infection. Black arrows indicate the positions of the IgG reactive SEA protein bands. Serum IgG of: **(A)** two mice (lanes 7 and 8) recognized the 73 and 78 kDa SEA bands at day 7 post-infection; **(B)** two mice (lanes 5 and 6) recognized the 73 and 78 kDa SEA bands at day 10 post-infection; **(C)** two mice (lanes 3 and 8) recognized the 121, 73 and 78 kDa SEA bands, and one mouse (lane 5) recognized the 121, 84, 73 and 78 kDa SEA bands at day 14 post-infection; **(D)** three mice (lanes 5, 6 and 7) recognized the 121, 73 and 78 kDa SEA bands, and two mice (lanes 8 and 9) recognized the 121, 84, 73, 78 and 55 kDa SEA bands at day 18 post-infection; **(E)** two mice (lanes 3 and 7) recognized the 121, 84, 73, 78 and 55 kDa SEA bands, two mice (lane 2 and 4) recognized the 121, 84, 73, 78, 55 and 47 kDa SEA bands, and three mice (lane 5, 8 and 9) recognized the 121, 73 and 78 kDa SEA bands at day 21 post-infection; **(F)** one mouse (lane 2) recognized the 121, 73 and 78 kDa SEA bands, six mice (lanes 3, 5, 7, 8, 9, 11) recognized the 121, 84, 73, 78 and 55 kDa SEA bands, and two mice (lanes 6 and 10) recognized the 121, 84, 73, 78, 55 and 47 kDa SEA bands at day 28 post-infection. Click here for file

Additional file 2 **Fig. S2 Immunoblotting profile of Sj23HD recognition by serum IgG of 10 individual mice infected with *S. japonicum *at different times**. For all blots, lane M, protein molecular weight markers; lane 1, no protein band was recognized by pooled sera of five mice before infection; lanes 2-11, sera of 10 individual mice on the indicated days post-infection. Black arrows indicate the position of IgG reactive Sj23HD protein band (33.5 kDa). The Sj23 HD protein was recognized by serum IgG of: **(A) **one mouse (lane 3) at day 7 post-infection; **(B) **one mouse (lane 8) at day 10 post-infection; **(C) **three mice (lanes 3, 4, 8) at day 14 post-infection; **(D) **five mice (lanes 4-8) at day 18 post-infection; **(E) **eight mice (lanes 2-9) at day 21 post-infection; **(F) **and all 10 mice (lanes 2-11) at day 28 post-infection. Click here for file

Additional file 3 **Fig. S3 Immunoblotting profile of SEA recognized by individual serum IgM of 10 individual mice infected with *S. japonicum *at different times post-infection**. For all blots, lane M, protein molecular weight markers; lane 1, no protein band was recognized by pooled sera of five mice before infection; lanes 2-11, sera of 10 individual mice at the indicated days post-infection. Black arrows indicate the positions of the IgM reactive SEA protein bands. Serum IgM of: **(A) **one mouse (lane 10) recognized the 73 and 78 kDa SEA bands at day 7 post-infection; **(B) **one mouse (lane 2) recognized the 73 and 78 kDa SEA bands, one mouse (lane 11) recognized the 73, 78 and 84 kDa SEA bands at day 10 post-infection; **(C) **three mice (lanes 2, 4 and 10) recognized the 121, 73, 78 and 55 kDa SEA bands at day 14 post-infection; **(D) **one mouse (lane 4) recognized the 55 and 78 kDa SEA band, one mouse (lane 6) recognized the 73 and 78 kDa SEA band, one mouse (lane 7) recognized the 121, 84, 73 and 78 kDa SEA bands, three mice (lanes 2 and 3) recognized the 121, 84, 73, 78 and 55 kDa SEA bands at day 18 post-infection; **(E) **one mouse (lane 3) recognized the 121, 84 and 73 kDa SEA band, four mice (lanes 2, 4, 5, 6) recognized the 121, 84, 73, 78 and 55 kDa SEA bands, and two mice (lanes 10, 11) recognized the 121, 84 kDa, 73 and 78 SEA bands at day 21 post-infection; **(F) **two mice (lanes 2, 4) recognized the 121, 73 and 78 kDa SEA bands, six mice (lane 3, 5, 6, 7, 8 and 9) recognized the 121, 84, 73 and 78 kDa SEA bands, and two mice (lane 10, 11) recognized the 121, 84, 73, 78 and 55 kDa SEA bands at day 28 post-infection. Click here for file

Additional file 4 **Fig. S4. Immunoblotting profile of Sj23HD recognized by serum IgM of 10 individual mice infected with *S. japonicum *at different times post-infection**. For all blots, lane M, protein molecular weight markers; lane 1, no protein band was recognized by pooled sera of five mice before infection; lanes 2-11, sera of 10 individual mice on the indicated days post-infection. Black arrows indicate the position of the IgM reactive Sj23HD protein band (33.5 kDa). The Sj23 HD protein was recognized by serum IgM of: **(A) **one mouse (lane 7) at day 7 post-infection; **(B) **one mouse (lane 5) at day 10 post-infection; **(C) **three mice (lanes 7, 8 and 11) at day 14 post-infection; **(D) **six mice (lanes 3-8) at day 18 post-infection; **(E) **nine mice (lanes 3-11) at day 21 post-infection; **(F) **and all ten mice (lanes 2-11) at day 28 post-infection. Click here for file
