# Supplementary figures and images for: Monitoring specific antibody responses against the hydrophilic domain of the 23 kDa membrane protein of Schistosoma japonicum for early detection of infection in sentinel mice
Source: Parasit Vectors. 2011 Sep 10;4:172. doi: 10.1186/1756-3305-4-172 (PMC3180346; doi:10.1186/1756-3305-4-172)

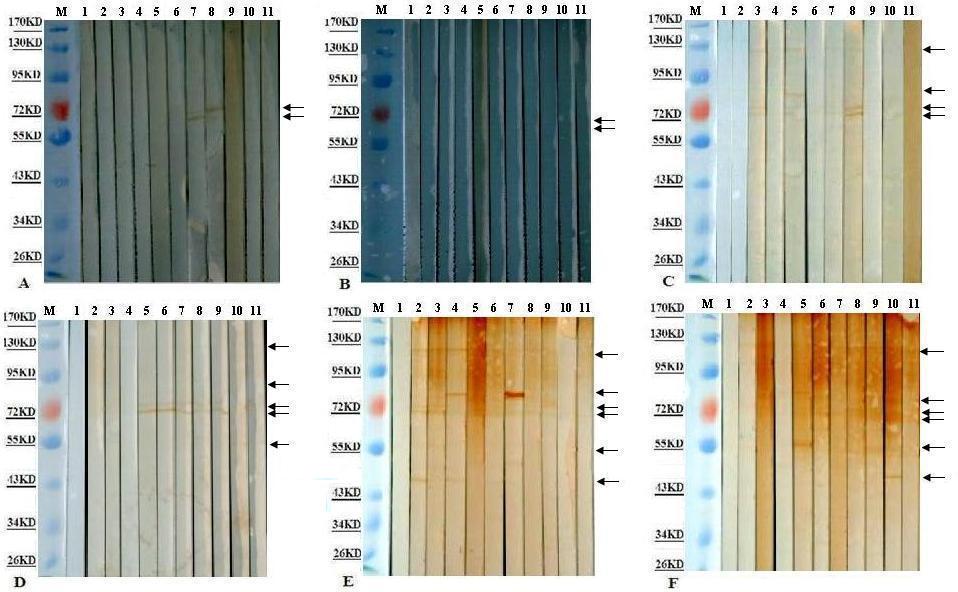

Supplement: Additional file 1 — Fig. S1 Immunoblotting profile of SEA recognition by serum IgG of 10 individual mice infected with S. japonicum at different times post-infection. For all blots, lane M, protein molecular weight markers; lane 1, no protein band was recognized by pooled sera of five mice before infection; lanes 2-11, sera of 10 individual mice on the indicated days post-infection. Black arrows indicate the positions of the IgG reactive SEA protein bands. Serum IgG of: (A) two mice (lanes 7 and 8) recognized the 73 and 78 kDa SEA bands at day 7 post-infection; (B) two mice (lanes 5 and 6) recognized the 73 and 78 kDa SEA bands at day 10 post-infection; (C) two mice (lanes 3 and 8) recognized the 121, 73 and 78 kDa SEA bands, and one mouse (lane 5) recognized the 121, 84, 73 and 78 kDa SEA bands at day 14 post-infection; (D) three mice (lanes 5, 6 and 7) recognized the 121, 73 and 78 kDa SEA bands, and two mice (lanes 8 and 9) recognized the 121, 84, 73, 78 and 55 kDa SEA bands at day 18 post-infection; (E) two mice (lanes 3 and 7) recognized the 121, 84, 73, 78 and 55 kDa SEA bands, two mice (lane 2 and 4) recognized the 121, 84, 73, 78, 55 and 47 kDa SEA bands, and three mice (lane 5, 8 and 9) recognized the 121, 73 and 78 kDa SEA bands at day 21 post-infection; (F) one mouse (lane 2) recognized the 121, 73 and 78 kDa SEA bands, six mice (lanes 3, 5, 7, 8, 9, 11) recognized the 121, 84, 73, 78 and 55 kDa SEA bands, and two mice (lanes 6 and 10) recognized the 121, 84, 73, 78, 55 and 47 kDa SEA bands at day 28 post-infection. [file 1756-3305-4-172-S1.JPEG]

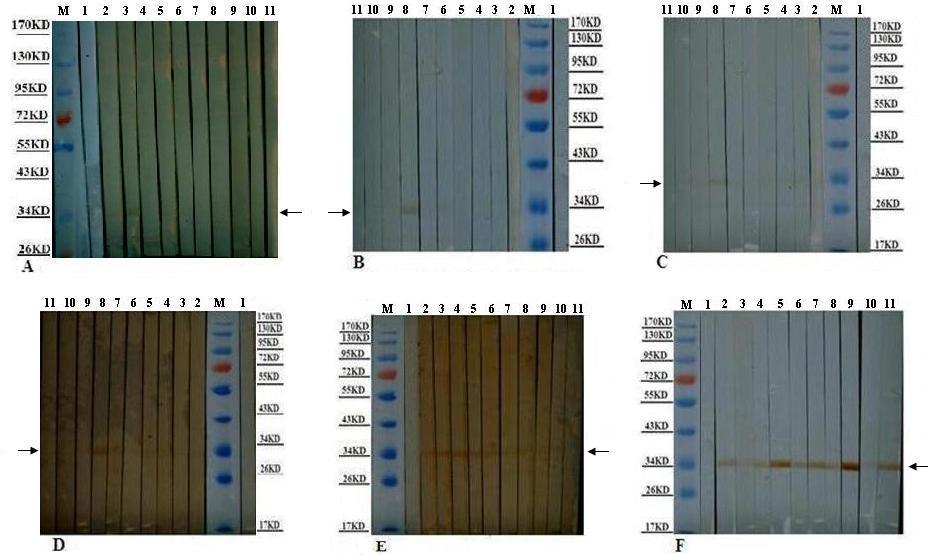

Supplement: Additional file 2 — Fig. S2 Immunoblotting profile of Sj23HD recognition by serum IgG of 10 individual mice infected with S. japonicum at different times. For all blots, lane M, protein molecular weight markers; lane 1, no protein band was recognized by pooled sera of five mice before infection; lanes 2-11, sera of 10 individual mice on the indicated days post-infection. Black arrows indicate the position of IgG reactive Sj23HD protein band (33.5 kDa). The Sj23 HD protein was recognized by serum IgG of: (A) one mouse (lane 3) at day 7 post-infection; (B) one mouse (lane 8) at day 10 post-infection; (C) three mice (lanes 3, 4, 8) at day 14 post-infection; (D) five mice (lanes 4-8) at day 18 post-infection; (E) eight mice (lanes 2-9) at day 21 post-infection; (F) and all 10 mice (lanes 2-11) at day 28 post-infection. [file 1756-3305-4-172-S2.JPEG]

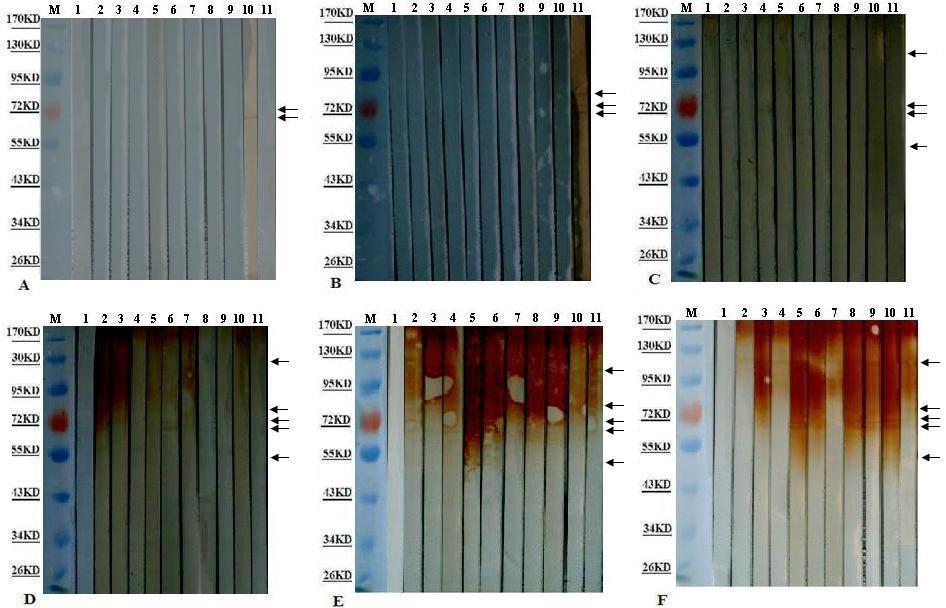

Supplement: Additional file 3 — Fig. S3 Immunoblotting profile of SEA recognized by individual serum IgM of 10 individual mice infected with S. japonicum at different times post-infection. For all blots, lane M, protein molecular weight markers; lane 1, no protein band was recognized by pooled sera of five mice before infection; lanes 2-11, sera of 10 individual mice at the indicated days post-infection. Black arrows indicate the positions of the IgM reactive SEA protein bands. Serum IgM of: (A) one mouse (lane 10) recognized the 73 and 78 kDa SEA bands at day 7 post-infection; (B) one mouse (lane 2) recognized the 73 and 78 kDa SEA bands, one mouse (lane 11) recognized the 73, 78 and 84 kDa SEA bands at day 10 post-infection; (C) three mice (lanes 2, 4 and 10) recognized the 121, 73, 78 and 55 kDa SEA bands at day 14 post-infection; (D) one mouse (lane 4) recognized the 55 and 78 kDa SEA band, one mouse (lane 6) recognized the 73 and 78 kDa SEA band, one mouse (lane 7) recognized the 121, 84, 73 and 78 kDa SEA bands, three mice (lanes 2 and 3) recognized the 121, 84, 73, 78 and 55 kDa SEA bands at day 18 post-infection; (E) one mouse (lane 3) recognized the 121, 84 and 73 kDa SEA band, four mice (lanes 2, 4, 5, 6) recognized the 121, 84, 73, 78 and 55 kDa SEA bands, and two mice (lanes 10, 11) recognized the 121, 84 kDa, 73 and 78 SEA bands at day 21 post-infection; (F) two mice (lanes 2, 4) recognized the 121, 73 and 78 kDa SEA bands, six mice (lane 3, 5, 6, 7, 8 and 9) recognized the 121, 84, 73 and 78 kDa SEA bands, and two mice (lane 10, 11) recognized the 121, 84, 73, 78 and 55 kDa SEA bands at day 28 post-infection. [file 1756-3305-4-172-S3.JPEG]

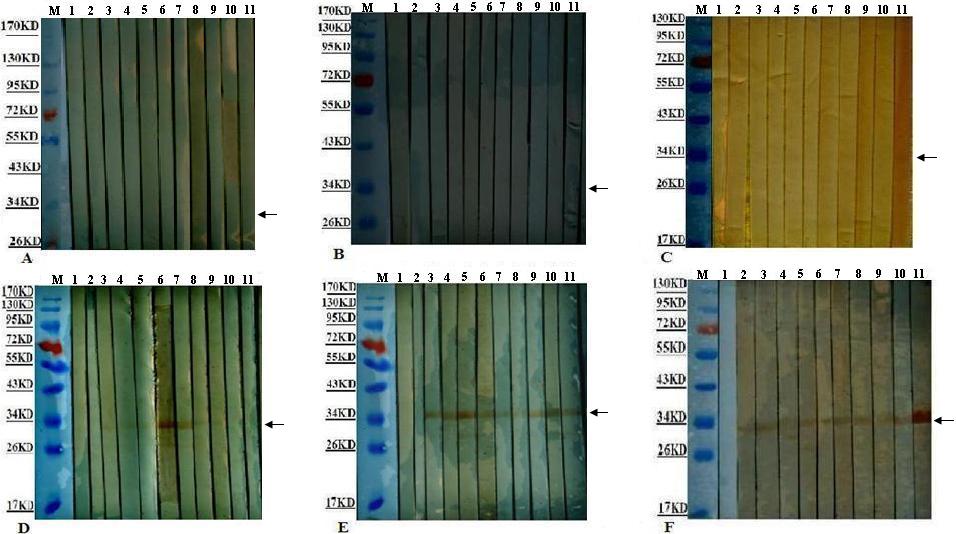

Supplement: Additional file 4 — Fig. S4. Immunoblotting profile of Sj23HD recognized by serum IgM of 10 individual mice infected with S. japonicum at different times post-infection. For all blots, lane M, protein molecular weight markers; lane 1, no protein band was recognized by pooled sera of five mice before infection; lanes 2-11, sera of 10 individual mice on the indicated days post-infection. Black arrows indicate the position of the IgM reactive Sj23HD protein band (33.5 kDa). The Sj23 HD protein was recognized by serum IgM of: (A) one mouse (lane 7) at day 7 post-infection; (B) one mouse (lane 5) at day 10 post-infection; (C) three mice (lanes 7, 8 and 11) at day 14 post-infection; (D) six mice (lanes 3-8) at day 18 post-infection; (E) nine mice (lanes 3-11) at day 21 post-infection; (F) and all ten mice (lanes 2-11) at day 28 post-infection. [file 1756-3305-4-172-S4.JPEG]
